# A sensitive one-pot ROA assay for rapid miRNA detection

**DOI:** 10.1007/s42994-024-00140-0

**Published:** 2024-03-18

**Authors:** Zhihao Hou, Wenpeng Deng, Alun Li, Ya Zhang, Jianye Chang, Xinyue Guan, Yuxiao Chang, Kaile Wang, Xinjie Wang, Jue Ruan

**Affiliations:** 1 Shenzhen Branch, Guangdong Laboratory of Lingnan Modern Agriculture, Genome Analysis Laboratory of the Ministry of Agriculture and Rural Affairs, Agricultural Genomics Institute at Shenzhen, Chinese Academy of Agricultural Sciences, Shenzhen, 518120 China; 2 Hubei Key Laboratory of Agricultural Bioinformatics, College of Informatics, Huazhong Agricultural University, Wuhan, 430070 China; 3 Department of Systems Biology, UT MD Anderson Cancer Center, Houston, TX 77030 USA

**Keywords:** miRNA, Rolling circle amplification (RCA), Sensitive detection, Fluorescence detection

## Abstract

MicroRNAs (miRNAs) and short RNA fragments (18–25 nt) are crucial biomarkers in biological research and disease diagnostics. However, their accurate and rapid detection remains a challenge, largely due to their low abundance, short length, and sequence similarities. In this study, we report on a highly sensitive, one-step RNA O-circle amplification (ROA) assay for rapid and accurate miRNA detection. The ROA assay commences with the hybridization of a circular probe with the test RNA, followed by a linear rolling circle amplification (RCA) using dUTP. This amplification process is facilitated by U-nick reactions, which lead to an exponential amplification for readout. Under optimized conditions, assays can be completed within an hour, producing an amplification yield up to the microgram level, with a detection limit as low as 0.15 fmol (6 pM). Notably, the ROA assay requires only one step, and the results can be easily read visually, making it user-friendly. This ROA assay has proven effective in detecting various miRNAs and phage ssRNA. Overall, the ROA assay offers a user-friendly, rapid, and accurate solution for miRNA detection.

**Supplementary Information:**

The online version contains supplementary material available at 10.1007/s42994-024-00140-0.

## Introduction

MicroRNAs (miRNAs) are endogenous non-coding single-stranded RNA molecules, with lengths ranging from 18 to 23 nts, and are ubiquitous in eukaryotic cells (Ha and Kim 2014). MiRNAs play a crucial regulatory role in many biological processes, including development, cell differentiation, apoptosis, proliferation, and immunity (Bartel 2018). Therefore, the ultra-sensitive detection of miRNAs is beneficial for many applications, in both clinical and non-clinical applications. However, the low levels of miRNAs in tissues and cells, their short length, and the high sequence similarity within miRNA families, make detection of miRNAs challenging (Dave et al. 2019).

Reverse transcription-quantitative polymerase chain reaction (RT-qPCR) is commonly used for detecting RNA, but it is not suitable for miRNA detection (Garibyan and Avashia 2013). Other miRNA analytical methods, such as Northern blot hybridization and microarray analysis, require specialized facilities and specific kits, and are therefore limited to laboratories (Lee et al. 2010; Várallyay et al. 2008).

Rolling circle amplification (RCA) is a simple and efficient isothermal enzymatic process (Zhao et al. 2008). The RCA primers are designed to detect target sequences, on a circular probe, using a strand displacement amplification enzyme (Phi29, Bst DNA polymerase) and generate long single-stranded DNA. The circular probe design of RCA has a powerful feature that enables exponential signal amplification, which significantly improves the sensitivity and dynamic range of the assay. RCA reactions have been used to develop various types of reactions, such as hyperbranched rolling cycle amplification (H-RCA) (Lizardi et al. 1998; Wang et al. 2005), multiple circle-to-circle rolling circle amplification (C2CA) (Dahl et al. 2004), and nicking-mediated rolling circle amplification (N-RCA) (Murakami et al. 2009). However, these expansion reactions require additional primers or specific reaction temperatures, and there is still room for improvement in specificity and ease of operation (Zhao et al. 2015).

In the current study, we developed the ROA assay for rapid and accurate miRNA detection. The method requires only a single reaction tube and isothermal incubation. ROA can be completed within an hour, producing easily detectable miRNA signals even without specialized equipment. Its exceptional sensitivity, with a detection limit as low as 0.15 fmol (6 pM), makes it ideal for analyzing even the most challenging miRNAs, such as miRNA299 and phage ssRNA. ROA represents a significant advancement in miRNA detection, offering researchers a powerful tool that is not only accurate and rapid but also remarkably user-friendly.

## Results

### Principle of the ROA

**Fig. 1:**
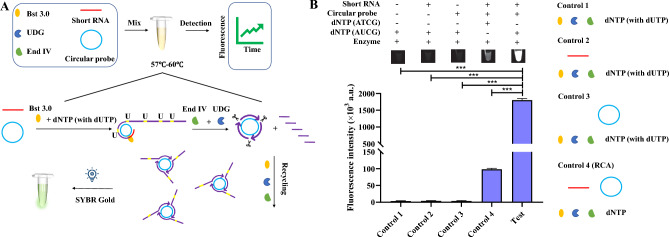
Principle of the ROA. **A** The reaction involves three principal steps: (1) The short RNA specifically hybridizes to the circularized probe in the presence of the enzyme mix; (2) the long sequence copy with dUTP of the template is synthesized by the Bst 3.0 polymerase; and (3) UDG and Endonuclease IV recognize the nicking sites and cleave these sequences. Multiple triggers are produced and initiate a new reaction cycle, and through multiple such reaction cycles, an exponential amplification for a small amount of RNA is achieved. **B** Controls employed in these experiments: Control 1, mixture without any nucleic acid; Control 2, mixture with only short RNA added; Control 3, mixture with only circularized probe added; Control 4, mixture with normal dNTP. Test: complete mixture

The principle of ROA is illustrated in Fig. 1. In the first step, a short RNA molecule hybridizes with a circular probe that then functions as a primer to initiate the ROA reaction, which is catalyzed by the Bst 3.0 polymerase. The reaction is maintained at a high temperature (57–60 °C) to prevent non-specific RNA from initiating the reaction. The products of the first step are long single-stranded DNA strands with a proportion of dUTP, which contains a candidate nicking site. The UDG and End IV enzymes are then used to nick the DNA, at the dUTP site, generating fragments of various lengths that can serve as second primers. Multiple rounds of amplification can then occur, as shown in Fig. 1A, by employing multiple primers that hybridize to the same circular probe, which was used in the first step. The high sensitivity of ROA allows for easy detection of the products, which can be visualized under blue excitation light or UV with the addition of SYBR Gold (Fig. 1B). Our analyses show that the ROA amplification efficiency is significantly higher than that of traditional RCA (control 4), and thus it represents a powerful new form of PG-RCA that has rapid, lower cost, and higher detection specificity.

### Optimization of the ROA conditions

**Fig. 2:**
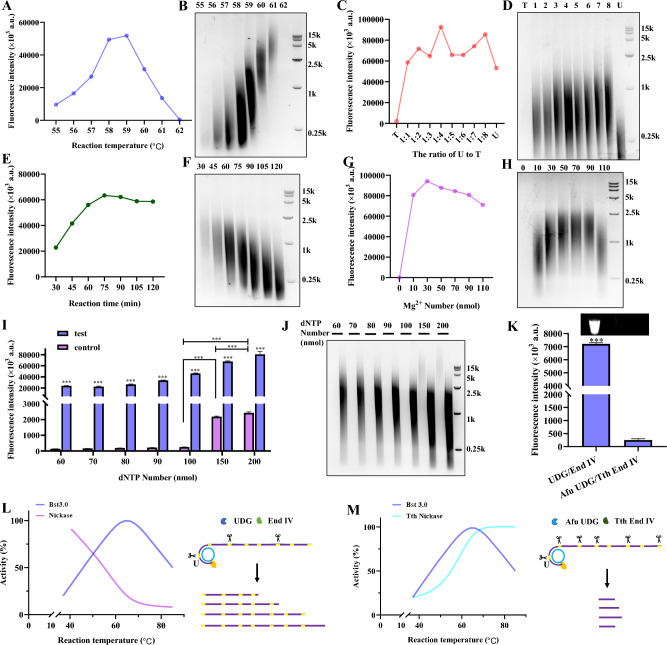
Optimization of the ROA conditions. Optimal reaction conditions for ROA tested by varying the reaction temperature (**A** and **B**), the ratio of U to T (**C** and **D**), reaction times (**E** and **F**), the Mg^2+^ concentration (**G** and **H**) and the dNTP concentration (**I** and **J**). The effects of different nickases on the reaction (**K**–**M**). The concentration of the test miRNA (miR165a) was 100 fmol. Fluorescence intensities were quantified using the Qubit ssDNA Assay Kit

The efficiency of ROA can be affected by various conditions, including reaction temperature, reaction time, U to T ratio, and magnesium ion concentration. Reaction temperature is a key factor in the ROA reaction (Liu et al. 2013), as it affects the enzyme activity and hybridization efficiency of nucleic acids. We quantitatively analyzed the effect of reaction temperature on ROA amplification with the Qubit ssDNA Assay Kit (Fig. 2A) and investigated the size of the products using agarose gel electrophoresis (Fig. 2B). The results from these assays indicated that the amplification product increased with the increase in reaction temperature from 55 to 59 °C, followed by a decrease above 59 °C, due to reduced End IV enzyme activity.

We further investigated the effect of the U to T ratio on the amplification efficiency of the ROA reaction (Fig. 2C, D). We found that the product of the ROA reaction was greatly increased compared to the RCA reaction (without dUTP), and the amplification efficiency was highest at a U to T ratio of 1:4. This may be because the dUTP is randomly added during the rolling amplification reaction, and the resultant fragment lengths, which vary based on the U to T ratio, are most suitable for annealing at the reaction temperature, thereby yielding the highest amplification efficiency.

Subsequently, we tested the effects of the ROA reaction times (Fig. 2E, F), the concentration of magnesium ions (Fig. 2G, H), and dNTP concentrations (Fig. 2I, J). Here we observed that the products of amplification increased with reaction times between 30 to 75 min. However, with reaction times longer than 75 min, the reaction product began to slowly decrease. The entire reaction system exhibited optimal efficiency at a magnesium ion concentration of 30 nmol (1.2 fM). Although high amplification efficiency was achievable at high dNTP concentrations, maintaining concentrations below 100 nmol (4 fM) prevented an increase in background signal.

For achieving high detection specificity and sensitivity, we capitalized on the distinctive activities of Bst 3.0 polymerase, UDG, and End IV (Fig. 2K**)**. Taking advantage of Bst 3.0 the polymerase enhanced activity at high temperatures, coupled with the low activity of UDG and End IV at these same high temperatures, resulted in a significantly increased amplification efficiency of the ROA, compared with the use of thermostable monofunctional Afu UDG and Tth End IV (Fig. 2L, M**).** Based on these optimized reaction conditions, we obtained a high sensitivity with a low detection limit and a wide dynamic range, which is comparable to PCR and PG-RCA.

### Sensitivity and specificity of the ROA

**Fig. 3:**
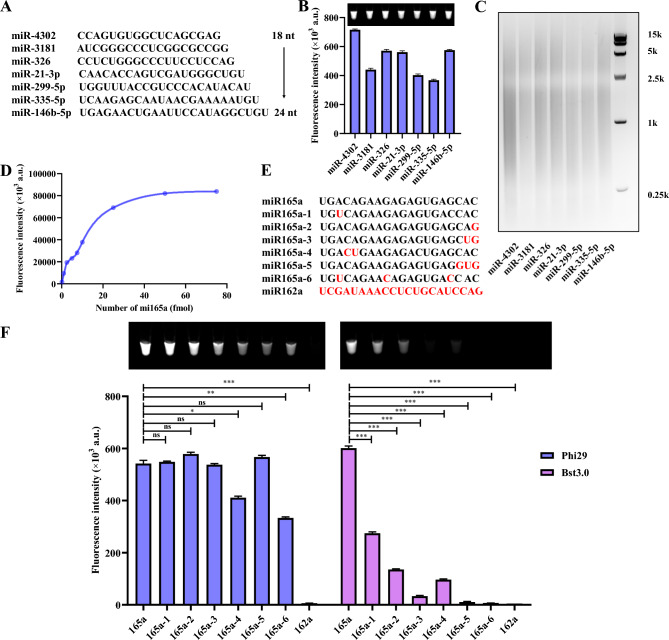
Sensitivity and specificity of ROA. **A** Test 18–24 nt miRNAs. **B** ROA analysis for the indicated 18–24 nt miRNAs. **C** Analysis of ROA product sizes for the indicated miRNAs.** D** The sensitivity of miR165a detection using ROA.** E** Sequences of miR165a, miR165a (1–6) and miR162a. **F** The specificity of miR165a detection using ROA and other PG-RCA

To ensure the precision of miRNA detection using the ROA assay, we conducted a comprehensive evaluation of its sensitivity and specificity. To further scrutinize its performance, we examined miRNAs spanning lengths from 18 to 24 nt (Fig. 3A), and the ROA assay demonstrated exceptional adaptability by successfully detecting miRNAs of all lengths (Fig. 3B, C). Then we tested the detection limit of the ROA assay. We used miR165a for testing, as depicted in Fig. 3D, the amplified product volume increased with the rise in miR165a concentration, ranging from 0.15 to 75 fmol, with the detection limit calculated to be 0.15 fmol (6 pM). The improved sensitivity of this method can be attributed to the extremely high amplification efficiency of RCA and multi-primer strand displacement amplification (MSA). Additionally, the large dynamic range of this ROA method (3 orders of magnitude) enables an accurate detection of miRNAs under various experimental conditions.

The specificity of a miRNA assay is crucial, particularly when detecting miRNA family members with high sequence homology. To this end, we tested the specificity of the ROA assay with synthesized miRNAs containing various nucleotide mismatches (Fig. 3E). At the recommended Bst 3.0 polymerase working temperature of 59 °C, non-homologous miR162a failed to be amplified. Next, we introduced single nucleotide mismatches, at different positions, and established that the ROA assay was sensitive to a single-nucleotide mismatch, with the largest reduction in amplification efficiency observed at the 3′ end. Double nucleotide mismatches were more sensitive than single-nucleotide mismatches, and the 3′ end (miR165a-3) was more sensitive than the 5′ end (miR165a-4) of the miRNA. Moreover, mismatches at three consecutive nucleotides at any place (miR165a-5 and miR165a-6) almost abolished the amplification (Fig. 3F). Under the same UDG-aided RCA, Phi29 polymerase is far inferior to Bst 3.0 polymerase in specificity. The Phi29 polymerase can only distinguish mismatches with large differences, and its ability to distinguish mismatches is weaker than that observed for Bst 3.0 under our imposed experimental conditions. This is because the Phi29 polymerase has an inherent 3′ → 5′ proofreading exonuclease activity, which can cut out the 3′ end mismatched bases and then the next match base starts the RCA reaction. These results clearly demonstrate that the ROA assay has high specificity that can discriminate between target and off-target miRNAs.

### Application of ROA in detecting miRNAs

**Fig. 4:**
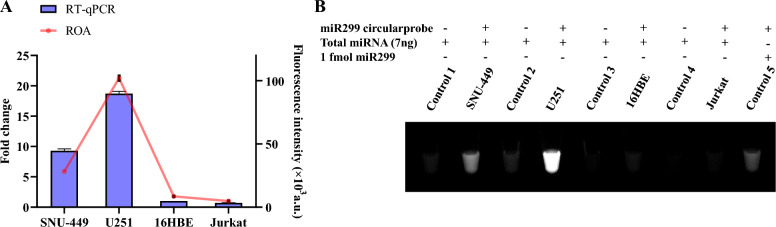
Application of ROA to detect miRNAs. **A** Comparison between the ROA and RT-qPCR for relative expression of miR299 in different cancer cell lines. **B** Fluorescent image analysis of ROA sample detection

To evaluate the efficacy of ROA in miRNA detection, we chose miR299 from different human cancer cell lines, including human hepatocellular carcinoma cell lines (SNU-449), human glioblastoma cell lines (U251), and an immortalized line of human T lymphocyte cells (Jurkat). Human normal bronchial epidermal cells (16HBE) were selected as the control sample. We extracted the cells, isolated their total miRNA, then detected the miRNAs using ROA. The ROA fluorescent detection system demonstrated that miR299 was selectively expressed in U251 and SNU-449 cells, which was consistent with the data from poly A-tailing-based RT–qPCR (Fig. 4).

### Applying ROA in detecting ssRNA Phage MS2

**Fig. 5:**
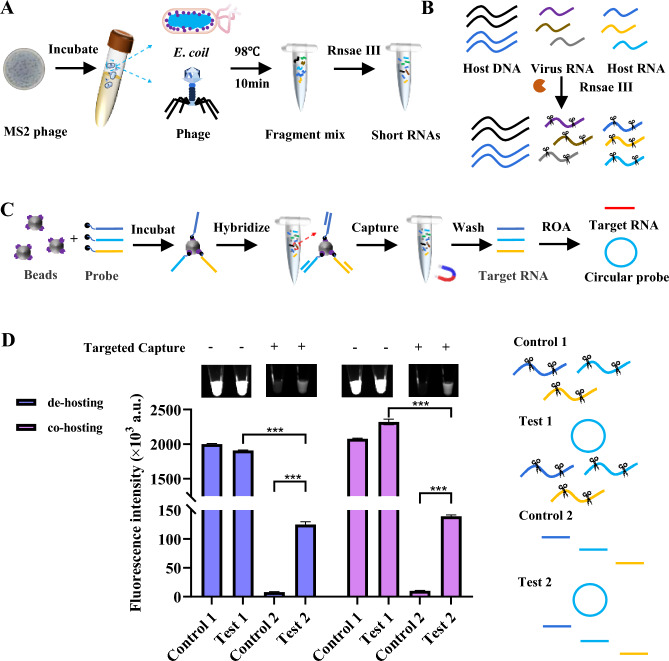
Employing ROA to detect ssRNA Phage MS2. **A** Simple fragmentation procedure of MS2 phage RNA. A host and phage co-culture was lysed, at high temperature, and the RNA was then fragmented with RNase III. **B** Nucleic acid composition after the simple phage and host treatment. Post-RNase III treatment, this system contained host DNA, fragmented host RNA and fragmented viral RNA. **C** Probe targeted capture process. Biotin-modified specific capture probes were combined with magnetic beads followed by incubation with the fragmented nucleic acids to capture viral RNA. Magnetic beads were then adsorbed to remove non-specific products, and captured fragments were eluted for ROA detection. **D** Histogram and fluorescent image depicting the ROA results for ssRNA phage MS2. After probe targeted capture, ROA specifically detect target RNA

A key disadvantage of laboratory-based virus detection systems is the requirement for specialized laboratory personnel and complex experimental equipment. This has led to a demand for rapid, real-time virus detection kits that are simple, reliable, affordable, and ultrasensitive. To test the ability of ROA to detect RNA viruses, a simple processing method was used to capture viral RNA. A lysis solution containing host cells and virus particles was subjected to a high temperature treatment to disassemble the viral particles (Fig. 5A**)**. The viral RNA was then fragmented, using RNase III, as it breaks the RNA into short fragments of 18–25 nucleotides, suitable for amplification using ROA (Fig. 5B**)**. A capture probe was designed using the flowchart in Fig. 5C, and the streptavidin probe method was used to efficiently capture the target RNA. This process can remove the interference of genomic DNA and host RNA and improve the specificity of ROA detection. Based on our test assays, we established that the simple capture of the sample to be tested can greatly reduce the non-specific amplification of the reaction (Fig. 5D). We could also demonstrate that magnetic bead capture was effective even without removing the host cells. These results demonstrate the clear potential of this ROA assay for the convenient and fast detection of viruses.

## Discussion

Among the many isothermal methods for miRNA detection, RCA is increasing in popularity for miRNA detection, due in part to its simplicity, specificity, and high sensitivity. To increase the sensitivity to the linear amplification limit of RCA, several methods of exponentiating RCA have been explored, including multi-primed RCA, which includes the addition of a second primer (Hutchison et al. 2005; Nilsson 2006), and PG-RCA using a nicking-endonuclease to produce targeted primers (Murakami et al. 2009, 2012). However, these methods still have the problem of cumbersome reactions. ROA is a one-step, one-pot isothermal assay that relies on the strand-displacing DNA synthesis by Bst 3.0 polymerase at higher temperatures. This ensures the specificity of the reaction compared with other UDG-aided RCA designs (Dong et al. 2019), as the polymerase is only active at high temperatures. Additionally, the clever use of UDG and End IV at higher temperatures is also an important factor in efficient ROA amplification.

High sensitivity is critical for detecting miRNAs in biological samples. ROA can directly use RNA as the primer for rolling circle amplification, the UDG and End IV can cleave the long ssDNA products, which in turn serve as secondary primers containing the target sequences to initiate subsequent RCA cycles, resulting in exponential amplification of the target. ROA has excellent sensitivity, with a detection limit of 0.15 fmol, and can be completed within an hour. ROA is a competitive alternative to existing isothermal amplification methods (Table 1). Concentrations of miRNA markers in biological fluids can be at or lower than pmol levels. Relevant to this work, the miR299-5p is considered as a potential biomarker in human hepatocellular carcinoma (Jiang and Shen 2018) and glioblastoma (Peng 2018; Zhong et al. 2022), and thus offer a promising route to explore disease-specific signatures.

**Table 1:** Comparison between ROA assay and similar molecular diagnostic methods

Methods	One-pot and One-step	RNA reverse-transcription	Detection time	Detection limit	References
RT-PCR	×	√	2 h	0.2 zmol	Chen et al. (2005)
RT-LAMP	√	√	1 h	1 zmol	Sun et al. (2017)
Cas12a-SCR	×	×	6 h	4.7 zmol	Wang et al. (2020)
HRCA	×	×	6 h	10 amol	Cheng et al. (2009)
C2CA	×	×	2 h	100 amol	Sanchez et al. (2021)
N-RCA	×	×	2 h	0.1 fmol	Xu et al. (2019)
ROA	√	×	1 h	0.15 fmol	This work

ROA is a promising new method for miRNA detection, but it has some limitations, one being the potential for non-specific amplification. Under certain conditions, template/primer-independent DNA synthesis can occur, via thermophilic DNA polymerase-mediated DNA synthesis (Hanaki et al. 1997; Hanaki et al. 1998; Liang et al. 2004). The non-specific reaction initiation in the negative control can be traced back to the dNTP substrates, DNA polymerase, and the nicking enzyme. The Bst 3.0 polymerase has been reported to polymerize dATP and dTTP to poly d(A-T), at a temperature range between 60 and 90 °C (Ogata and Miura 1998), and this reaction is exacerbated by the presence of dUTP and nickase (Fig. S1A). To avoid non-specific amplification, it is important to optimize the reaction conditions. Our findings indicated that a high dNTP concentration (100 nmol) can readily cause background interference, especially when the quantity of nickase (UDG 5 U/μL and End IV 10 U/μL) surpasses that of the Bst 3.0 polymerase (8 U/μL) (Fig. S1B). A reduction in dNTP concentration to 10 nmol mitigates background interference, and further reducing it to 5 nmol ensures an interference-free reaction, under these conditions. This is a relatively safe parameter to proceed with the reaction.

In summary, the ROA assay stands out as a single-step process that operates without the need for specialized equipment. The results are easily visualized by the naked eye, rendering it user-friendly and showcasing its potential for integration into a fully automated portable system for on-site diagnostics.

## Methods

### Circular probe design

The design rules for constructing the circular probe are shown in Fig. S2. The host RNA was first cut into 16-mers, which are fragments consisting of 16 consecutive bases. An overlap of 15 bases was used between adjacent 16-mers. Each 16-mer was then converted to a 32-bit integer and stored in a bitmap for ease of use on a personal computer. Through this process we created an index that is 512 MB in size for each host RNA sequence, and the index can contain multiple host RNA sequences at the same time without an increase in size. The index can be stored for future use.

The viral mRNA sequence was cut into a series of 20-mers, with an overlap of 19 bases between adjacent segments. Each 20-mer was again cut into five 16-mers and then each 16-mer was checked to see if it was present in the host index. Only the 20-mer for which none of the five 16-mers was present in the host index was considered valid. For each valid 20-mer, GC content, annealing temperature, 3′-end specific base, and homopolymer checks were performed. The 20-mers were then joined head-to-tail in pairs. The 16-mer at the junction was checked to see if it was present in the host index. If it was, the joining order between the two 20-mers was declared invalid. All 20-mers that matched the valid joining order were used as the alternate 20-mers to construct the circular probe. Four 20-mer positions among the alternative 20-mers that were more evenly distributed in the viral mRNA were selected to be annealed first. This was done so that the order of the adjacent 20-mers matched the order in the alternative 20-mer. Finally, the reverse complementary sequence of the circular probe was generated, as well as the corresponding reverse complementary sequences of the four 20-mers that make up the circular-probe. The program’s source code can be downloaded from https://github.com/dwpeng/roa.

### Circular probe preparation

The following protocol was used to ligate 80–100 bp single-stranded DNA (ssDNA) (Table S1): 1 μL of ssDNA (100 ng/μL) was mixed with 2 μL of CircLigase™ 10 × Reaction Buffer (CL4111K), 1 μL of CircLigase™ ssDNA Ligase (100 U), 1 μL of ATP (1 mM), 1 μL of MnCl_2_, and 14 μL of H_2_O. The mixture was incubated at 60 °C for 4 h, then at 25 °C for 1 h. The enzymes were inactivated by heating the mixture at 95 °C for 3 min, then immediately placing it on ice for 2 min. Then 1 μL of Exonuclease I (NEB, M0293S) and 0.5 μL of Exonuclease III (NEB, M0206S) were added to the reaction, and incubated at 37 °C for 2 h. The mixture was purified using ZYMO DNA Clean & Concentrator (D4029), and the circular probe concentration was quantified using Qubit ssDNA Assay Kit (Q10212). Polyacrylamide (10%) gel electrophoresis (PAGE) was performed in 1 × TBE buffer (9 mM boric acid, 0.2 mM EDTA, 9 mM Tris–HCl, pH 7.9) at 180 V and room temperature for 60 min. The gel was stained with gel red and visualized using a GelView 6000 Plus Imaging System (Fig. S3).

### miRNA synthesis and preparation

The synthetic miRNA sequences (Table S2) were purchased from Shanghai Sangon Biological Engineering Technology & Services. The oligonucleotides and miRNAs were diluted with RNase-free ddH_2_O to 10 μM for the preparation of stock solutions. The stock solutions were denatured at 95 °C for 1 min and then immediately placed on ice for 3 min. The denatured solutions were ready for use in subsequent experiments. Using Qubit microRNA Assay Kits (Q32880) to measure the concentration, we calculated the number of moles based on DNA length and concentration using Eq. 1.

1 $$\frac{\mathrm{\mu g DNA}}{{\text{mL}}}\times \frac{{\text{mL}}}{1000\mathrm{ \mu L}}\times \frac{{\text{pmol}}}{330\mathrm{ pg}}\times \frac{10^6\mathrm{ pg}}{1\mathrm{ \mu g}}\times \frac{1}{{\text{N}}}=\frac{\mathrm{pmol DNA}}{\mathrm{\mu L}}$$

N is the number of nucleotides and 330 pg/pmol is the average molecular weight of a nucleotide. Gradient dilutions were then obtained from the calibrated concentration.

### Rolling circle amplification

The following reagents were mixed in a 25 μL reaction mixture: 0.5 μL circularized DNA (1 ng), 1 μL RNA primers, 1 μL Bst 3.0 Polymerase (8,000 U/μL) (NEB, M0374L), 0.5 μL Uracil-DNA Glycosylase (UDG) (NEB M0280S), 0.5 μL Endonuclease IV (NEB M0304S), 1 μL 25–90 mM dNTP mix (including dUTP) (N0446S), 2.5 μL rCutSmart Buffer (B6004S), 18 μL H_2_O. The reaction mixture was incubated at 58–60 °C for 60–90 min. The concentration of the linearized DNA was measured using the Qubit ssDNA Assay Kit.

### Quantitative real-time PCR detection

Total miRNAs from SNU-449, U251, Jurkat, and 16HBE were obtained from the Chinese Academy of Sciences (Shanghai, China). For this procedure we used, 20 μL of the RT containing 10 μL of 2 × miRNA RT Mix (Vazyme, MR201), 2 μL of HiScript miRNA Enzyme Mix, 100 ng–2 μg of miRNA and water. The RT system was incubated at 37 °C for 60 min and then inactivated at 85 °C for 5 min. The cDNA solution was diluted by 3 × before being added to the qPCR system which contained 10 μL of 2 × ChamQ Universal SYBR qPCR Master Mix (Vazyme, Q711), 0.8 μL of the PCR primer, 1 μL of cDNA template and 8.2 μL of water. The temperature program consisted of 95 °C for 2 min, 40 cycles of 95 °C for 10 s, and 60 °C for 30 s.

### Phage amplification and lysis

*Escherichia coli* strain C-3000 and Phage MS2 were purchased from ATCC. For bacterial growth, a single colony of *E. coli* strain C-3000 was inoculated into 15 mL nutrient broth (NA) and incubated at 37 °C overnight with shaking. 5 μL of the overnight culture was transferred to a new tube and incubated with shaking at 37 °C until an optical density (OD_600nm_) of 0.5–1 was reached. A 15 mL aliquot of the culture was transferred to a new tube to be used as the bacterial growth control.

For phage infection, 20 μL of phage MS2 suspension was added to 10 mL of the grown bacterial culture. The phage-mediated infection proceeded with shaking at 37 °C for 4–5 h (Luzon-Hidalgo et al. 2021). A 50 μL aliquot of the co-culture was added to NA broth and placed in a PCR tube. The sample was denatured at 98 °C for 10 min, and then immediately place on ice.

### RNA fragmentation and specific probe capturing

The following protocol was used for RNA fragmentation: a 5 µL aliquot of 10 × ShortCut Reaction Buffer was mixed with 5 µL of ShortCut RNase III (NEB M0245), 5 µL of 10X MnCl_2_, and 35 µL of the lytic phage used in the previous step. The sample was incubated for 20 min at 37 °C, then added 10 µL 10 × EDTA to stop the reaction.

### RNA isolation using streptavidin magnetic beads

A 12.5µL Streptavidin Magnetic Beads (NEB S1420) was added to a clean RNase-free microcentrifuge tube, and 100 µL of Wash/Binding Buffer (0.5 M NaCl, 20 mM Tris–HCl, 1 mM EDTA) was added and the beads then washed twice. A 10 µL aliquot of biotin-probe (100 mM) solution was added to magnetic beads and vortexed to suspend the beads. The mixture was then incubated at room temperature for 5 min, and the supernatant was then discarded. A 100 µL aliquot of Wash/Binding Buffer was then added and beads were washed, twice. Next a 50 µL aliquot of the sample containing the multiple RNA fragments was added to the prepared magnetic beads, vortexed to suspend the particles, and then incubated at room temperature for 10 min. A magnetic field was applied, and the supernatant was removed after adding a 100 µL aliquot of Wash/Binding Buffer, and washed, twice. A 100 µL aliquot of cold Low Salt Buffer (0.15 M NaCl, 20 mM Tris–HCl, 1 mM EDTA) was added to the beads, vortexed to suspend the beads, and a magnetic field was applied to remove and discard the supernatant. A 25 µL aliquot of 70 °C prewarmed Elution Buffer (10 mM Tris–HCl, 1 mM EDTA) was then added, vortexed to suspend beads, and then incubated at room temperature for 2 min. A magnet field was applied to separate the beads from solution, and then the supernatant was transferred to a clean RNase-free microcentrifuge tube, and finally a 25 µL aliquot of fresh Elution Buffer was added to obtain the purified RNA fragments.

### Fluorescence measurement and gel electrophoresis

SYBR Gold Nucleic Acid (Invitrogen, S11494) was diluted tenfold with TE buffer. One microliter of the diluted solution was added to the reaction system and the reaction mixture was then imaged with ultraviolet (UV) light under the GelView 6000 Plus Imaging System. The SYBR Gold-labeled DNA could also be visualized, by the naked eye, using blue excitation light at a wavelength of 470 nm.

Agarose (1.2%) gel electrophoresis was carried out in 1 × TAE buffer (20 mM acetic acid, 1 mM EDTA, 40 mM Tris–HCl, pH 7.6) at 160 V and room temperature for 60 min.

## Supplementary Information

Below is the link to the electronic supplementary material.

Supplementary file1 (DOCX 919 KB)

## Data Availability

All data supporting the findings of this study are available within the article and Supplementary Files. The ROA source code is hosted by GitHub at: https://github.com/dwpeng/roa.
