# Supplementary material for: A sensitive one-pot ROA assay for rapid miRNA detection
Source: aBIOTECH. 2024 Mar 18;5(3):298–308. doi: 10.1007/s42994-024-00140-0 (PMC11399362; doi:10.1007/s42994-024-00140-0)
Supplement: Supplementary file 1 — Supplementary file1 (DOCX 919 KB) [file 42994_2024_140_MOESM1_ESM.docx]

**Supplementary Figures**


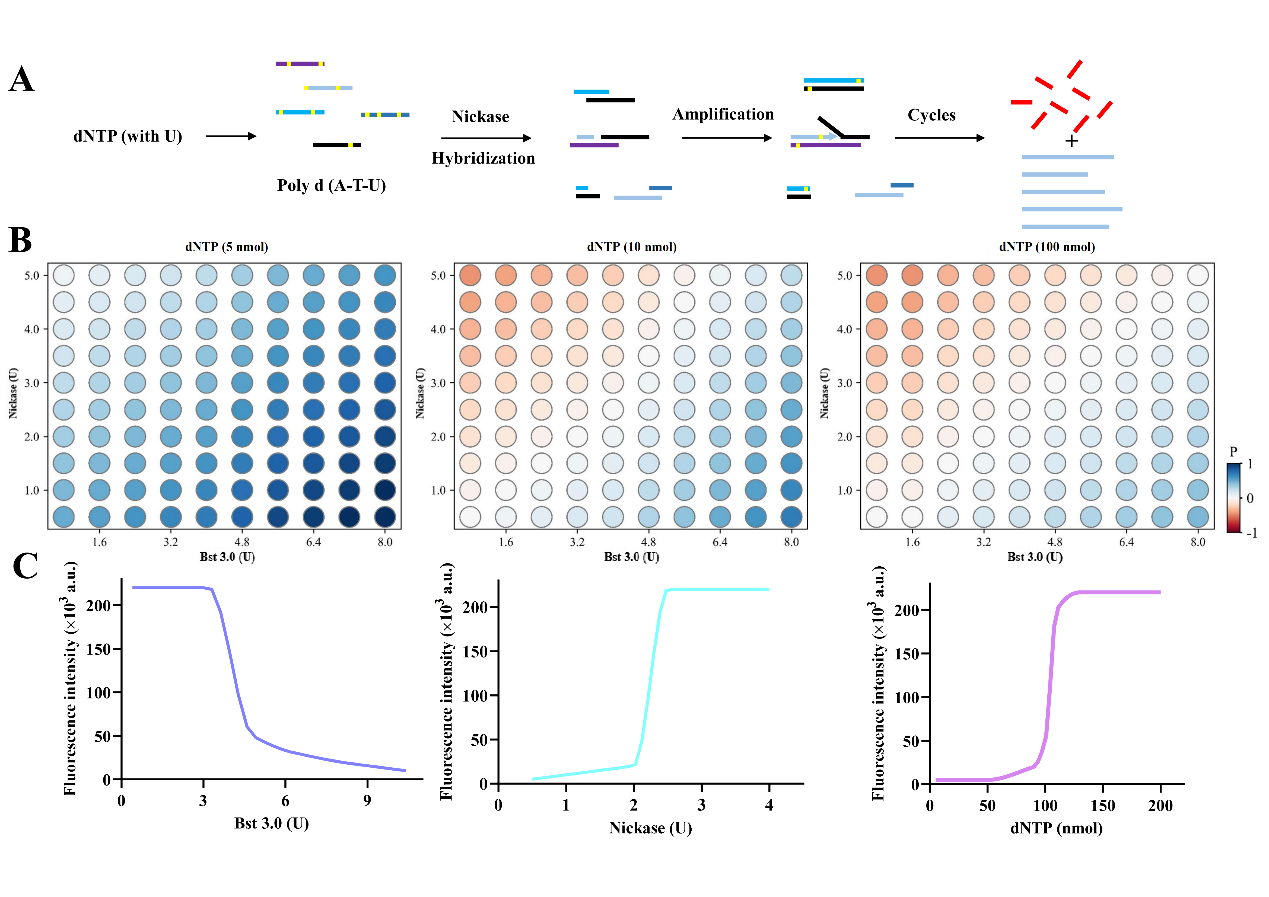


**Fig. S1** Minimizing background signal. Each reaction system contained only dNTP, Bst, UDG or End IV, without any nuclease. **A** Mechanism of background signal amplification using the ROA. **B** Heat map of background interference at three different dNTP concentrations. The color intensity represents the strength of background interference. **C** The emergence of background interference under three restrictive conditions; background signal amplifications were suppressed under the following reaction conditions: Bst 3.0 > 4U; Nickase < 2.5U; dNTP <100 nmol.


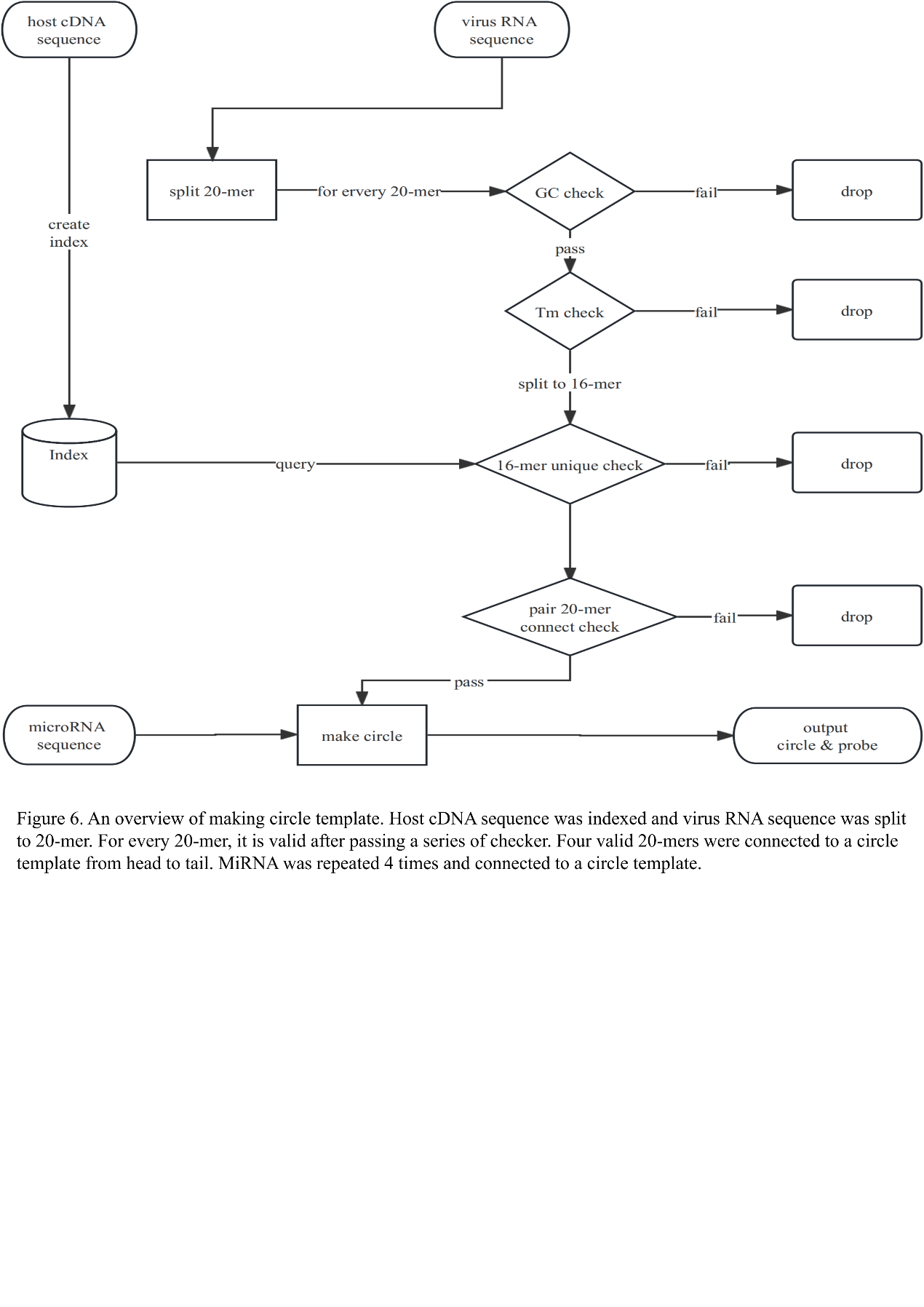


**Fig. S2** Schematic overview of the procedure employed to make circle templates. The host cDNA sequence was first indexed, and the viral RNA sequence was then split into 20-mers. Each 20-mer was validated after passing a series of checks. Four valid 20-mers were connected to a circular template, from head to tail. The miRNA sequence was repeated four times and connected to a circular template.

**
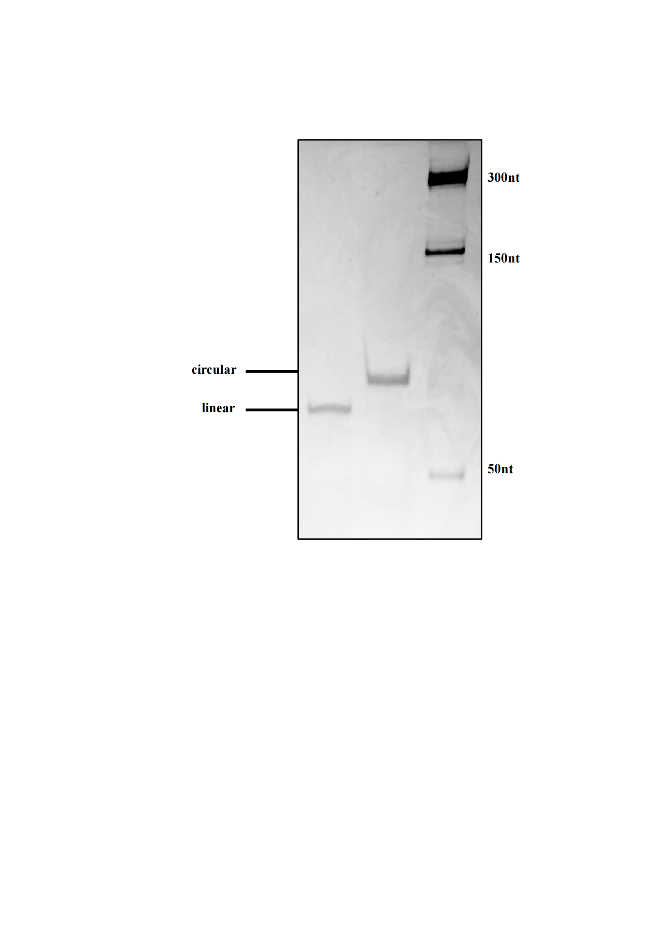
**

**Fig. S3** PAGE image of linear and circular probes.

**Table S1.** Sequences of the oligonucleotides employed in this study.

| Probe identity | Sequence (5’-3’) |
| --- | --- |
| miR165a circular probe | pGTGCTCACTCTCTTCTGTCAGTGCTCACTCTCTTCTGTCAGTGCTCACTCTCTTCTGTCAGTGCTCACTCTCTTCTGTCA |
| miR4302 circular probe | pCTCGCTGAGCCACACTGGCTCGCTGAGCCACACTGGCTCGCTGAGCCACACTGGCTCGCTGAGCCACACTGG |
| miR3181 circular probe | pCCGGCGCCGAGGGCCCGATCCGGCGCCGAGGGCCCGATCCGGCGCCGAGGGCCCGATCCGGCGCCGAGGGCCCGAT |
| miR326 circular probe | pCTGGAGGAAGGGCCCAGAGGCTGGAGGAAGGGCCCAGAGGCTGGAGGAAGGGCCCAGAGGCTGGAGGAAGGGCCCAGAGG |
| miR21-3p circular probe | pACAGCCCATCGACTGGTGTTGACAGCCCATCGACTGGTGTTGACAGCCCATCGACTGGTGTTGACAGCCCATCGACTGGTGTTG |
| miR299-5p circular probe | pATGTATGTGGGACGGTAAACCAATGTATGTGGGACGGTAAACCAATGTATGTGGGACGGTAAACCAATGTATGTGGGACGGTAAACCA |
| miR335-5p circular probe | pACATTTTTCGTTATTGCTCTTGAACATTTTTCGTTATTGCTCTTGAACATTTTTCGTTATTGCTCTTGA |
| miR146b-5p circular probe | pACAGCCTATGGAATTCAGTTCTCAACAGCCTATGGAATTCAGTTCTCAACAGCCTATGGAATTCAGTTCTCA |
| miR4302/3181/335/146b  circular probe | pCTCGCTGAGCCACACTGGCCGGCGCCGAGGGCCCGATACATTTTTCGTTATTGCTCTTGAACAGCCTATGGAATTCAGTTCTCA |
| miR122 circular probe | pCAAACACCATTGTCACACTCCACAAACACCATTGTCACACTCCACAAACACCATTGTCACACTCCACAAACACCATTGTCACACTCCA |
| miR21-5p/299 circular probe | pTCAACATCAGTCTGATAAGCTAATGTATGTGGGACGGTAAACCATCAACATCAGTCTGATAAGCTAATGTATGTGGGACGGTAAACCA |

Note: The p in the circular temple indicates a 5′ phosphate modification.

**Table S2.** Sequences of miRNAs employed in this study.

| Probe identity | Sequence (5’-3’) |
| --- | --- |
| miR165a | UGACAGAAGAGAGUGAGCAC |
| miR4302 | CCAGUGUGGCUCAGCGAG |
| miR3181 | AUCGGGCCCUCGGCGCCGG |
| miR326 | CCUCUGGGCCCUUCCUCCAG |
| miR21-3p | CAACACCAGUCGAUGGGCUGU |
| miR299-5p | UGGUUUACCGUCCCACAUACAU |
| miR335-5p | UCAAGAGCAAUAACGAAAAAUGU |
| miR146b-5p | UGAGAACUGAAUUCCAUAGGCUGU |
| miR122 | UGGAGUGUGACAAUGGUGUUUG |
